# Inferring behavioural states from tracking data with hidden Markov models – a validation study using GPS video-camera collars

**DOI:** 10.1186/s40462-025-00621-x

**Published:** 2026-01-24

**Authors:** Benjamin Larue, Jonathan J. Farr, Libby Ehlers, Jim Herriges, Torsten Bentzen, Michael J. Suitor, Kyle Joly, Théo Michelot, Barbara Vuillaume, Steeve D. Côté, Eliezer Gurarie, Mark Hebblewhite

**Affiliations:** 1 Wildlife Biology Program, Department of Ecosystem and Conservation Sciences, University of Montana, Missoula, MT USA; 2 Bureau of Land Management, Fairbanks, AK USA; 3 Alaska Department of Fish and Game, Fairbanks, AK USA; 4 Department of Environment, Yukon Government, Whitehorse, Yukon Territory Canada; 5 National Park Service, Yukon-Charley Rivers National Preserve, Fairbanks, AK USA; 6 Department of Mathematics and Statistics, Dalhousie University, Halifax, NS Canada; 7 Département de biologie, Caribou Ungava and Centre d’études nordiques, Université Laval, Québec, Canada; 8 Laboratoire de Biométrie et Biologie Evolutive, Centre National de Recherche Scientifique, UMR 5558, Université Lyon I, Villeurbanne, France; 9 Department of Environmental Biology, State University of New York - College of Environmental Science and Forestry, Syracuse, NY USA

**Keywords:** Hidden Markov model, Wildlife tracking, Movement ecology, Caribou, GPS video collar, Temporal scale, Animal behaviour

## Abstract

**Background:**

Hidden Markov models (HMMs) are increasingly used to infer animal behavioural states from GPS tracking data, yet their interpretation often remains uncertain in the absence of empirical validation. Misinterpretation of statistical states as biologically meaningful behaviours can undermine scientific understanding and conservation decisions. Our objective was to evaluate how well HMM-inferred states correspond to directly observed behaviours and to test how the temporal resolution of GPS sampling influences behavioural inference.

**Methods:**

We used GPS collars equipped with video cameras to validate HMM-inferred behavioural states in 81 female migratory caribou (*Rangifer tarandus*) from two herds. We compared states derived from two- and three-state HMMs to behaviours observed in short collar video clips. To assess the effect of temporal scale, we fit HMMs to GPS data resampled at 20-, 60-, and 120-minute relocation intervals.

**Results:**

HMM-inferred behavioural states frequently diverged from video-observed behaviours at the start of observed GPS steps, especially at longer relocation intervals. These mismatches appeared to result from overlapping movement metrics among caribou behaviours (e.g., foraging vs. resting or traveling) and the inability of coarser GPS data to capture behavioural switches occurring at finer temporal scales than the fix rate. Videos of eating were the most misaligned with HMM-inferred states, likely due to high variation in caribou movement while foraging that is often characteristic of mixed-feeding large herbivores. Inferred states for a given location were often inconsistent across temporal scales, indicating that HMM outputs must be interpreted cautiously with respect to the GPS sampling frequency.

**Conclusions:**

The predicted HMM state can differ substantially from true behaviour at the start of each step, in particular at coarse temporal scales. Our results serve as a reminder to interpret HMM states over whole steps rather than at observed positions, validate movement-derived states where possible, and align sampling resolution with species-specific behavioural patterns.

**Supplementary Information:**

The online version contains supplementary material available at 10.1186/s40462-025-00621-x.

## Background

The rapid expansion of high-resolution animal tracking technology has revolutionized movement ecology, enabling researchers to investigate how animal movements relate to eco-evolutionary processes across spatial and temporal scales [1–3]. In parallel, a growing suite of statistical tools has been developed to infer latent behavioural states (or “modes”; [4]) from movement metrics derived from GPS data including behavioural change point analysis [5] and state-space models [4], within which hidden Markov models (HMMs; [6]) represent a widely used subclass. These tools now allow ecologists to address behaviourally focused questions solely from telemetry data, opening the door to important applied conservation research. For example, identifying the timing and location of foraging [7], parturition [8, 9], or responses to anthropogenic disturbance [10] can inform conservation strategies and help delineate critical habitats of high functional value [11–13].

Among these tools, HMMs have emerged as particularly popular due to their mathematical tractability, flexibility, and accessibility [6, 14, 15]. HMMs are commonly used to derive an underlying sequence of latent states (interpreted as behaviours) from a time series of observed animal movement metrics (e.g., step lengths and turn angles; [6, 15]). The latent process can take on a user-specified number of states, each with its own distribution of movement metrics. HMMs also provide state occupancy probabilities/assignments and state transition probabilities that can capture animal behavioural dynamics [7]. It is common practice to interpret these states post hoc using expert knowledge, usually based on stereotyped patterns in the state-dependent distributions —for example, short step lengths with tortuous paths (uniform distribution of turn angles) may be interpreted as foraging or resting, while long, directed steps may be classified as travelling [16–18]. Crucially, HMM states are often assigned to observed GPS locations; typically, the state for a relocation from *t* to *t* + 1 is viewed as a proxy for the animal’s behaviour at location *t*. This is for example implied whenever GPS locations are coloured by HMM states for visualisation, or when HMM outputs are used to investigate spatial distributions of animal behaviours (e.g [19]).

Despite their widespread use in movement ecology, HMMs are often applied without formal validation against independently observed behaviours. This raises concerns about the ecological validity of the interpretation of HMM-inferred behavioural states and their suitability for informing management decisions. A growing but still limited number of studies have begun to address this gap. For example, time–depth recorders were used to indirectly validate a foraging state in northern gannets (*Morus bassanus*; [20]), while biosonar recordings were matched to HMM states in Mexican fish-eating bats (*Myotis vivesi*; [21]). HMM-inferred behaviours were also compared to direct field observations in African lions (*Panthera leo*; [22]) and terns (*Sterna* spp.; [23]). These studies illustrate that while validation is feasible, it remains rare and often relies on species-specific technologies or assumptions. Furthermore, the effect of temporal scale on HMM-based behavioural inferences has yet to be evaluated. Without validation and careful interpretation, HMM-based inferences may produce misleading state assignments, ultimately undermining their utility for ecological inference and conservation planning [24].

As highlighted in previous studies [25, 26], careful consideration of behavioural repertoires, temporal sequences, and model setup is essential to avoid such pitfalls. Equally important, however, is the recognition that temporal resolution fundamentally shapes what behavioural dynamics can be detected. As Johnson [27] argued in his hierarchical framework of habitat selection, the spatial scale of observation determines which ecological processes are visible and interpretable. By analogy, the temporal scale of GPS tracking could influence whether HMMs capture fine-scale behavioural switches or only broad movement modes. Despite this, the sensitivity of HMM-derived states to temporal resolution remains largely untested.

We used a promising technology, GPS video-camera collars, to compare direct video-observed behaviours to HMM-inferred behavioural states across contrasting contexts. GPS video-camera collars provide a unique opportunity for validation of wild, remote and/or particularly elusive species as they simultaneously collect GPS relocations needed to fit HMMs and short videos from which reference behaviours can be easily observed [28–30]. Using data from 81 GPS video-camera collared female migratory caribou (*Rangifer tarandus*), we addressed three main questions: (1) How consistently do HMM-inferred behavioural states match reference caribou behaviours observed in collar videos in two- vs. three-state models?, (2) How does the temporal scale of GPS relocations (20 min vs. 60 min vs. 120 min) affect matching of HMM states to video behaviours?, and (3) To what extent does the temporal scale of GPS relocation intervals influence HMM-inferred behavioural state assignments?

In this study, we intentionally adopted a simple HMM implementation, not as a recommended best practice, but as a test case to demonstrate how misleading inferences can arise when critical considerations of behavioural repertoires, temporal scales, and validation are overlooked. By framing our analysis this way, our goal is to highlight the risks of naive HMM application and to encourage more deliberate integration of ecological knowledge, methodological rigor, and consideration of temporal scale when applying HMMs to animal tracking data.

## Methods

### Study areas and data collection

**Fig. 1:**
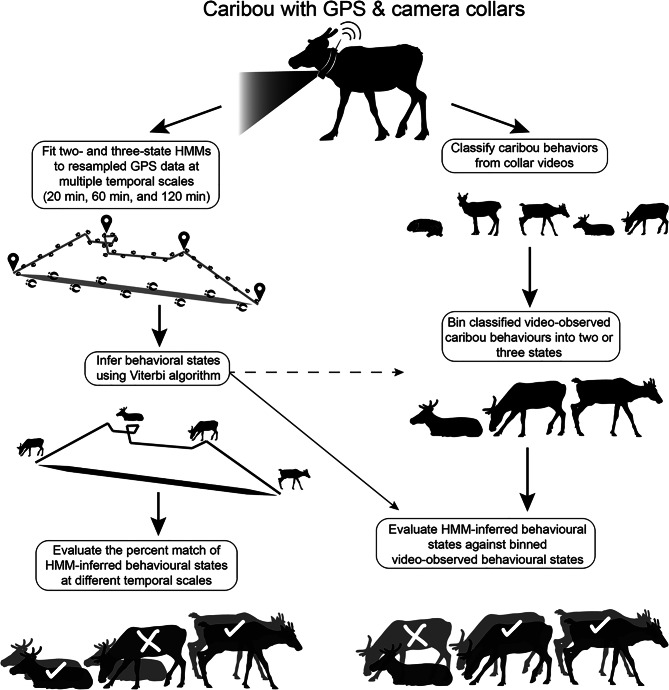
Flowchart representing the sequential steps we took to evaluate migratory caribou behavioural states inferred from hidden Markov model (HMM) using GPS video-camera collars. We specifically evaluated the sensitivity of HMM behavioural inferences to the temporal scale of GPS tracking (bottom left) and how well HMM-inferred behavioural states matched-up to 9 or 10-second video-observed caribou behaviours at given GPS locations (bottom right). Artwork by Kira Cassidy

Our study areas consisted of the summer range of the Fortymile caribou herd (FMCH) in east-central Alaska, United States, and north-central Yukon, Canada, and the summer range of the Rivière-aux-Feuilles caribou herd (RFCH) in the Ungava Bay of Québec, Canada. Using netguns or tranquilizer darts from a helicopter, 30 adult female caribou from the FMCH were captured during March and April 2018 and 2019 [31] and 51 adult female caribou from the RFCH at the end of March or beginning of April 2016, 2017, and 2018 [32]. During captures, we fitted each caribou with a GPS-Iridium collar integrated with a video camera and programmed to drop off on 10 September of the respective study year for FMCH females and 1 September for RFCH females. Because video footage could not be collected in darkness, collars were programmed to operate only during daylight hours. Consequently, both videos and GPS relocations were restricted to daylight periods, and no night-time data were available for analysis. During daylight hours, FMCH video collars recorded a 9-second video and a time-matched GPS relocation every 20 min, whereas RFCH video collars recorded a 10-second video every 20 min and a GPS relocation every 60 min. To evaluate how the temporal scale of GPS relocation intervals affects inferences from HMMs (see *HMM-derived behavioural inferences* below), we resampled GPS data to obtain sequences of consecutive GPS relocations at 20-min, 60-min, and 120-min intervals for the FMCH, and 60-min and 120-min intervals for the RFCH (Fig. 1). When resampling to coarser intervals, only every *n*th location was retained (e.g., every third fix when resampling from 20 to 60 min), and the intervening locations were dropped. This procedure ensured that the set of retained locations was identical across temporal scales, allowing direct comparison of behavioural states inferred at the same relocations but under different temporal resolutions. See Fig. 1 for a methodological flowchart. We conducted all analyses in the software R version 4.4.0 [33], and resampled GPS data using the track_resample function in package *amt* [34].

### Video-derived behavioural classification

For the FMCH video data, trained volunteers classified female caribou behaviours from a random subset of 18,134 videos out of the total of 175,504 videos as described in Ehlers et al. [31]. In this subset, caribou behaviours were coded into mutually exclusive categories, meaning that each video was assigned a single dominant behaviour. The categories included: eating (actively consuming vegetation), ruminating (chewing cud), travelling (directed movement while walking or running), stationary awake (alert but not moving or feeding), napping (resting in a laying position), and other (behaviours not fitting into the above categories).

In addition, a locomotion state was also coded for each FMCH video, which referred only to the movement of the animal rather than the finer behaviour it was performing (e.g., whether it was feeding or vigilant). Locomotion states were therefore limited to: napping, stationary awake, running, wading/swimming, and walking. These categories are mutually exclusive and were later used in our re-binning procedure to align video observations with HMM-derived states.

For the RFCH video data, trained personnel classified female caribou behaviours in 50,963 videos out of the total of 195,462 videos as described in Béland et al. [32]. Behaviours were coded into the following categories: eating (actively consuming vegetation), foraging (searching for and handling food, often while moving), drinking, laying (resting in a recumbent position), walking, ruminating, vigilant (alert scanning), running, swimming, and other (behaviours not fitting into the above categories). For the RFCH, video classification was not mutually exclusive, meaning that a caribou could be classified as ruminating and laying in the same video, for instance.

### HMM-derived behavioural inferences

We used HMMs to identify behavioural states from FMCH and RFCH individual GPS sequences. We fitted HMMs to estimate non-observable latent states (behaviours) based on the observed step lengths and turn angles in GPS time series of consecutive relocations using the moveHMM package [15]. Step lengths were calculated in kilometres as the linear distance of relocations (between locations *t* and *t* + 1, *t* + 1 and *t* + 2, etc.), while turn angles (in radians) were calculated from the angle between sets of three consecutive locations (two relocations, e.g., *t* and *t* + 1 and *t*-1, etc.). We did not account for individual variation in movement by fitting mixed-effect HMMs because the inclusion of individual random effects rarely improves HMM state assignment relative to fixed effects models [35]. After visually inspecting histograms of movement parameters, we used gamma distributions to model step length and von Mises distributions for turning angles. The moveHMM package performs maximum likelihood estimation and is somewhat sensitive to the initial values for the step length distribution parameters used to maximize the likelihood [14, 15]. To avoid converging on local maxima, we therefore randomly generated 10 sets of starting parameters from a range of values chosen based on visual examination of histograms of step length and turn angle distributions. We kept the model with the highest log-likelihood [7]. From our fitted HMMs, we decoded behavioural states using the Viterbi algorithm, which assigns the most likely state sequence based on estimated parameters [14, 15]. We also estimated state-dependent distributions of step lengths and turn angles.

We fitted separate two- and three-state HMMs to GPS sequences that were resampled to various relocation intervals (see above *study area and data collection*). HMMs can include more than three states, but models with fewer states are more easily associated with caribou behaviours of interest. Furthermore, the biological meaning of two or three behavioural movement modes for large herbivores has been explored by previous studies and few empirical studies aim to discriminate more than three behavioural states [7, 36].

### Comparing video- and HMM-inferred behavioural states

Each state in the Viterbi sequence strictly corresponds to a movement step (with a given step length and turning angle), but it is commonly interpreted as a proxy for the animal’s behavioural state at the start point of the step (e.g., to define spatial distributions of behaviours). Here, we adopt the latter interpretation to showcase how it can lead to misleading results. Importantly, this comparison between HMM states and video-derived behaviours is not intended as a full validation of the HMMs but rather as a limited check, given that HMMs classify behaviour over the entire step interval rather than at its starting point. This comparison therefore illustrates that video observations taken at specific relocations can only partially inform the interpretation of GPS-based HMM states, and that these states should not be interpreted as instantaneous behaviours at the moment a step begins. After behaviourally interpreting HMM results (see HMM-inferred behavioural states in the Results section below), we binned video behaviours into two or three broad categories to align with the HMM classifications (Fig. S7).

For the FMCH dataset, behaviours were coded into mutually exclusive categories, meaning each video was assigned a single dominant behaviour. For comparison to the two-state HMM models, we grouped videos classifications based on locomotion status: (1) *napping* and *stationary awake* were classified as “stationary”, and (2) *running*, *wading/swimming*, and *walking* were classified as “travelling”. For the three-state HMM models, we grouped videos classifications according to behavioural categories: (1) *ruminating*, *stationary awake*, and *napping* were classified as “stationary”, (2) *eating* was classified as “foraging”, and (3) *travelling* was classified as “travelling”.

For the RFCH dataset, behaviours were not mutually exclusive, so a single video could carry multiple labels. To compare with two-state HMMs, we applied a deterministic precedence rule: if any moving behaviour (*walking*, *running*, *swimming*) was present, the video was classified as “travelling”, overriding concurrent stationary labels. If no moving behaviour was present but at least one other behaviour (*eating*, *drinking*, *laying*, *ruminating*, *vigilance*, *foraging*) was present, the video was classified as “stationary”. For the three-state RFCH classification, we applied a hierarchical precedence rule. Observations were first assigned to “stationary” if any low-movement behaviours (*laying*, *vigilance*, *ruminating*) were present. If any locomotion behaviours (*walking*, *running*, *swimming*) occurred, the observation was reassigned to “travelling”. Finally, if any feeding behaviours (*eating*, *drinking*, *foraging*) occurred, the observation was overridden and classified as “foraging”. This rule ensured that co-occurrences such as *walking* with *eating* or *foraging* were assigned to “foraging”, while locomotion without feeding defaulted to “travelling”, and non-feeding stationary behaviours defaulted to “stationary”. For both FMCH and RFCH, we excluded all videos with the ambiguous behavioural classification “other” from our analyses.

**Fig. 2:**
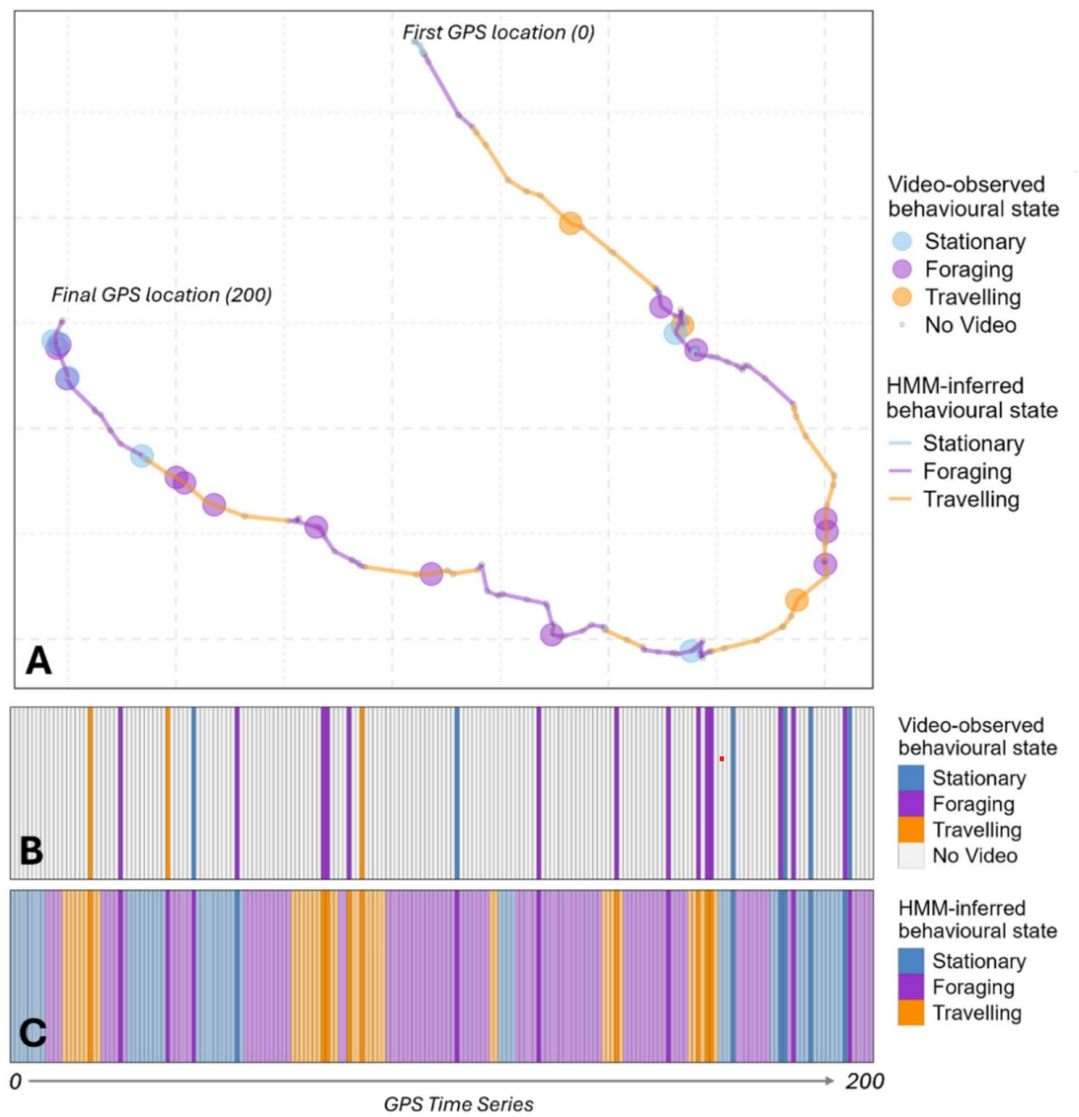
Comparison of hidden Markov model (HMM)-inferred and video-observed three-state behavioural classifications from Fortymile caribou herd GPS video-camera collar data, resampled to 120-minute fix intervals. This example showcases 200 consecutive GPS relocations from a randomly selected individual. Panel (**A**) displays the GPS track color-coded by HMM-inferred behavioural states with larger dots indicating the behavioural states identified from video observations at specific relocations. No video (small dots) indicates that the collar video was not behaviourally classified. Panels (**C**) and (**D**) present the corresponding sequences of HMM-inferred and video-observed behavioural states over time, facilitating direct visual comparison between model inferred states and direct video observations. Note that while HMMs are fit on GPS relocation data, HMM viterbi states were assigned to the first location (t) of each pair of locations (t, t + 1) per relocation interval to allow for clear comparison to videos at corresponding locations

After binning video-observed behaviours into two or three broader categories to match HMM-inferred states (Fig. 2 and Figs. S1–S3), we evaluated classification performance using confusion matrices with the caret R package [37], where the reference labels were the video-derived states and the predictions were the HMM-inferred states. From these, we calculated the overall accuracy (percent match), defined as the proportion of observations where the HMM state matched the corresponding video category.

We assessed accuracy across a total of ten situations: all combinations of two- vs. three-state HMMs with relocations every 20 min, 60 min, or 120 min for the FMCH, and all combinations of two- vs. three-state HMMs with relocations every 60–120 min for the RFCH.

### Sensitivity to temporal scale

To evaluate the sensitivity of inferred HMM behavioural states to the temporal scales of GPS relocation intervals, we compared behavioural states inferred from two- and three-state HMMs fitted to the same GPS data resampled every 20 min, 60 min, or 120 min. That is, for each comparison, we down-sampled the finer-scale state sequence to match the time resolution of the coarser state sequence. To quantify agreement, we calculated the percent match (overall accuracy), defined as the proportion of locations where the behavioural state inferred at one temporal scale matched the state inferred at another temporal scale. For the FMCH we quantified six match percentages for comparisons of behavioural states inferred at three temporal scales (20 min vs. 60 min vs. 120 min) for two- and three-state HMMs. For the RFCH we quantified two match percentages for comparisons of behavioural states inferred at two temporal scales (60 min vs. 120 min) for two- and three-state HMMs. In addition, we calculated the average time in minutes that individuals spent in each behavioural state before switching at the different relocation intervals, separately for each herd, to further illustrate how apparent behavioural bout lengths based on HMMs varied across temporal scales. In other words, for each herd and relocation schedule we estimated the mean duration of consecutive runs of a given state (number of consecutive locations multiplied by the relocation interval), providing a complementary metric of how temporal resolution influences the perceived length of behavioural bouts.

## Results

### HMM-inferred behavioural states

For both the FMCH and RFCH, two-state HMMs consistently identified distinct movement patterns at all temporal scales (Figs. S1-S2). The first state was characterized by shorter step lengths and low directionality (i.e., weakly concentrated turn angles), indicative of localized movement or stationarity with apparent movements caused by GPS error. The second state exhibited longer step lengths with strongly concentrated turn angles near zero, reflecting highly directional movement. Based on the literature, the first state corresponds to a behavioural state typically identified as “foraging”, “resting” or “resident”, while the second state represents what has often been inferred to be “relocations” or “transient” movements [36]. Accordingly, we nominally classified these statistical states as “stationary” and “travelling”, respectively.

In three-state models, the movement patterns were less clearly differentiated (Figs. S3-S4). Across both herds and all temporal scales, we identified a first state with shorter step lengths and weakly concentrated turn angles, a second state with intermediate step lengths and moderate turn angle concentration, and a third state with longer step lengths and highly directional movement. Based on these distributions, we nominally classified the state with shortest step lengths as “stationary”, the state with intermediate step lengths and some tortuosity as “foraging”, and the third state, characterized by long, directed movements, as “travelling” [7].

### Comparing video- and HMM-inferred behavioural states

**Fig. 3:**
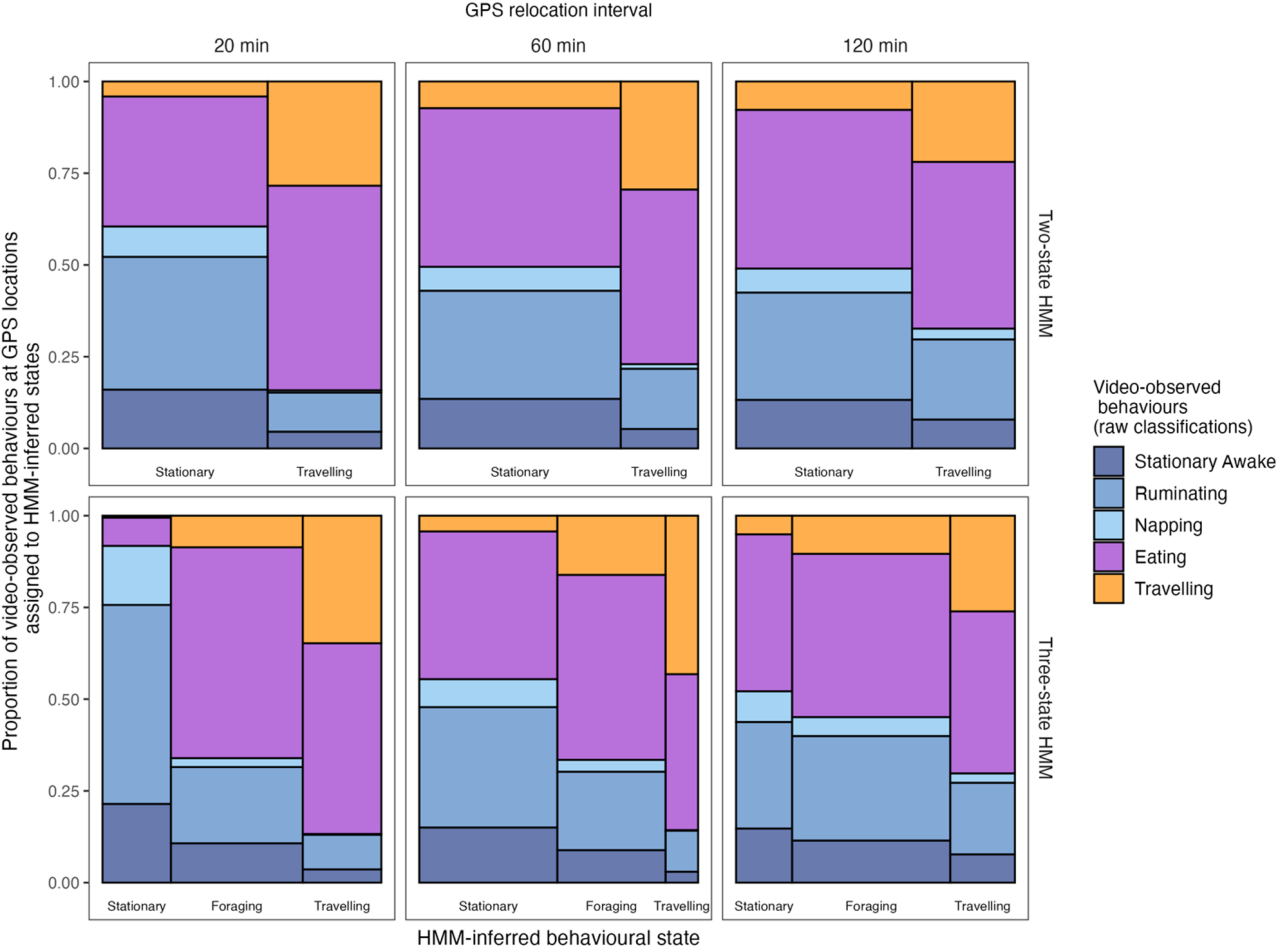
Proportion of raw classifications of Fortymile caribou video-observed behaviours in each inferred hidden Markov model (HMM) Viterbi behavioural state in two-state models (top row) and three-state models (bottom row) at GPS locations resampled to different GPS relocation intervals (from left to right: 20 min, 60 min, and 120 min). HMM behavioural states were matched to video-observed behaviours at the starting location of a relocation interval. The width of columns is proportional to the number of GPS locations associated to each Viterbi behavioural state (stationary, foraging, or traveling)

**Fig. 4:**
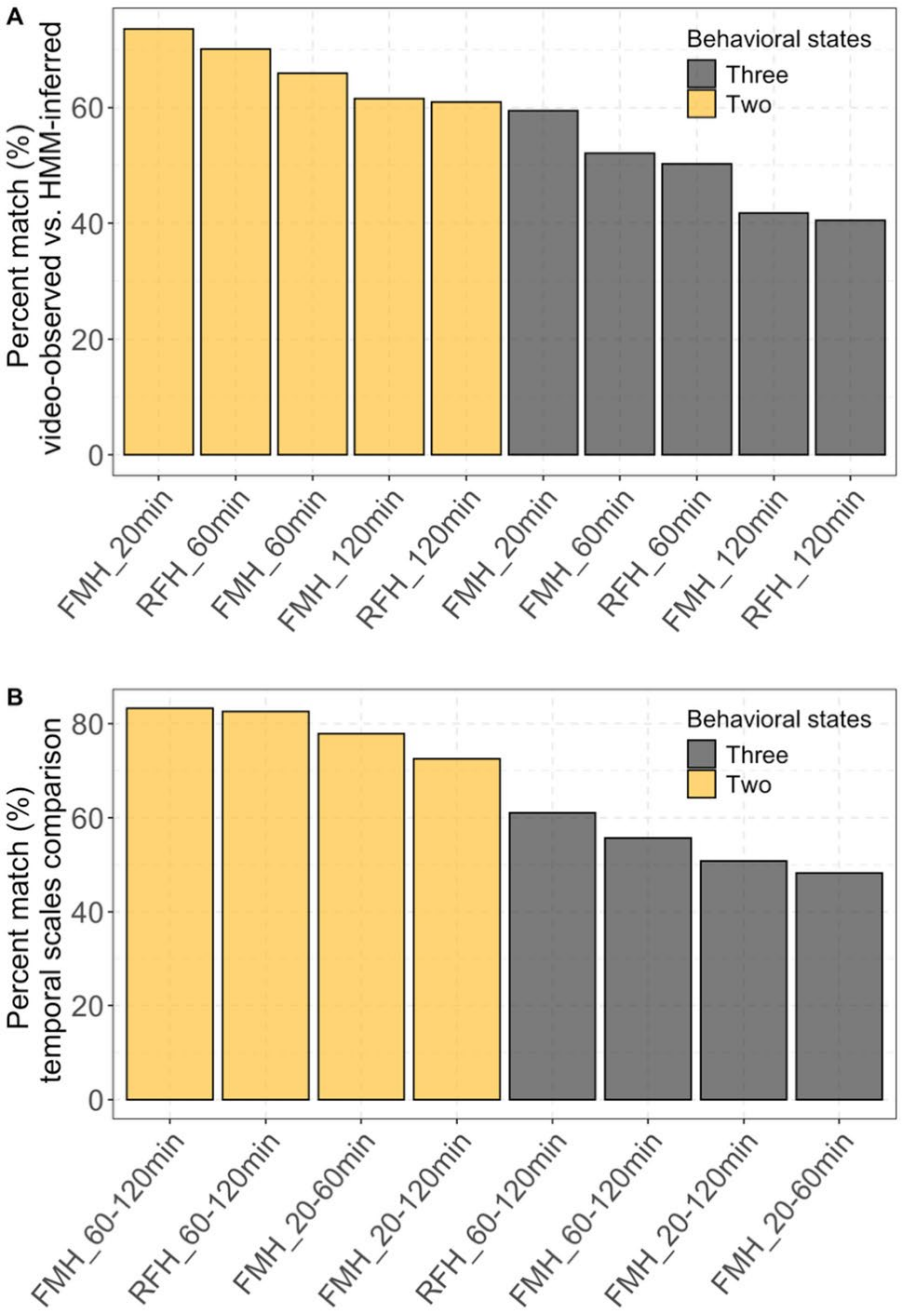
Evaluation of behavioural states inferred from two- (gold) and three-state (grey) hidden Markov models (HMMs) based on the movement path metrics of female migratory caribou from the Fortymile (FMCH) and Rivière-aux-Feuilles (RFCH) herds at different temporal scales (20 min, 60 min, or 120 min). Panel (**A**) presents the percent match of HMM-inferred behavioural states against reference behavioural states observed from video-camera collars. Panel (**B**) presents the percent match among behavioural states inferred from HMMs fitted to GPS data resampled to different temporal scales. For example, in Panel (**B**), the gold bar over FMCH 60–120 min shows the percent match of behavioural states inferred from two-state HMMs fitted to Fortymile caribou GPS data with relocation intervals of 60 min vs. 120 min. HMM states were assigned to the starting location (t, not t + 1) for each relocation interval to be compared to videos and resampled GPS data at 20, 60 and 120-minute intervals

Our results revealed that the HMM-inferred caribou behavioural states frequently did not match reference video-observed behavioural states at the start of the GPS steps, in both two- and three-state HMMs across temporal scales and herds (Figs. 3 and 4a; Figs. S5-6). For example, for the two-state models, HMM-inferred behavioural states differed from video-derived states 26.4–39.0% of the time, depending on the GPS relocation interval and herd (Fig. 4a). We found that behavioural inferences from two-state models matched video-observed behavioural states more frequently than those from three-state models (Fig. 4a). We also found that HMM-inferred behavioural states obtained from shorter GPS relocation intervals matched video-observed states more frequently than HMM-inferred states obtained from longer GPS relocation intervals (Fig. 4a; Figs. S5-6). Indeed, inferences from the FMCH two-state HMM fitted to 20-minute relocation interval data validated best against video-observed behaviours with a 73.6% match (Fig. 4a). On the other hand, the three-state HMMs resampled to 120-minute relocation intervals had the lowest matching percentage, with only 41.8% and 40.5% of HMM-derived behavioural states matching video-observed states for the FMCH and RFCH, respectively.

Nonetheless, some notable patterns emerged (Fig. 3; Table S1). In the FMCH three-state HMM, for example, only between 0.5 (at 20 min) to 5.1% (at 120 min) of the steps classified as “stationary” by the HMM started from a point where the animal was travelling according to the video data. Similarly, across all the three-state HMMs almost no napping occurred in the “foraging” and “travelling” states. There was much less eating in the “stationary” state at 20 min intervals, than in either of the other states (7.7% at 20 min vs. 40.2% of 60 min and 42.8% at 120 min). In contrast, 54.2% of the 20 min “stationary state” consisted of rumination.

### Sensitivity to temporal scale

Our results indicate that the temporal scale of GPS tracking (20 min vs. 60 min vs. 120 min) had a considerable effect on HMM-derived behavioural state inferences. We found that the inferred behaviour at the start point of a step frequently depended on the temporal scale (Fig. 4b). Therefore, HMM states tentatively interpreted as “stationary”, “foraging”, and “traveling” behaviours did not match at different temporal scales, highlighting the need for scale-specific behavioural inference. Indeed, most comparisons had over 20% mismatches (average mismatch of 20.9% for two-state models and 46.0% for three-state models; Fig. 4b). For both two-state and three-state HMMs, the comparison between 60-minute relocation intervals vs. 120-minute relocation intervals yielded the highest match percentages for both herds (Fig. 4b). Conversely, FMCH behavioural inferences from 20-minute relocations vs. 60-minute relocations were less aligned (Fig. 4b). Consistent with these findings, our analysis of the average time spent in each state before switching showed that apparent bout lengths increased substantially with coarser relocation intervals (Table 1). For example, in the FMCH, two-state models indicated that “stationary” bouts increased from 168 min at 20-min intervals to 760 min at 120-min intervals, while “traveling” bouts rose from 364 min to 655 min. Similar patterns were evident in three-state models and for the RFCH, where average bout lengths nearly doubled between 60- and 120-minute relocations. These results demonstrate that temporal scale strongly shapes not only state classification at individual relocations but also the inferred duration of behavioural bouts, with coarser scales inflating estimates of time spent in each state.

**Table 1:** Average time (minutes) spent in each behavioural state before switching, inferred from two- and three-state HMMs at different GPS relocation intervals (20, 60, and 120 min) for the fortymile caribou herd (FMCH) and the Rivière-aux-Feuilles caribou herd (RFCH)

		Two-state HMM		Three-state HMM		
Herd	Relocation interval	Stationary	Travelling	Stationary	Foraging	Travelling
FMCH	20 min	168	364	56	75	110
	60 min	481	269	256	172	209
	120 min	760	655	745	733	641
RFCH	60 min	281	208	175	153	177
	120 min	464	448	502	431	426

## Discussion

Model validation studies are essential to assess the reliability of model-based inferences across realistic contexts. However, validation studies can be challenging, especially for the behaviours of remotely tracked animals which are dependent on the measurement tools used and the time frames over which they generate inference. Here, we attempted a partial validation of hidden Markov models (HMMs) as a tool to infer animal behavioural states at GPS tracking positions across two- vs. three-state models, three temporal scales, and two migratory caribou herds. By leveraging the unique advantages of GPS video-camera collars, we found that HMM-inferred caribou behavioural states frequently did not match reference video-observed behaviours at the start of movement steps. We also showed that our HMM behavioural inferences are sensitive to the temporal scale of GPS tracking. These mismatches also revealed important insights into the complexity and nuanced nature of caribou behaviours within broader HMM movement states, providing valuable understanding about behavioural variability. Importantly, our intention was not to present an optimized, best-practice implementation of HMMs, but rather a deliberately simple application designed to demonstrate how misleading inferences can arise when validation and key considerations identified are overlooked [25, 26]. Our findings underscore the need for caution and critical reflection when interpreting behavioural states from HMM analyses at specific GPS locations, rather than over whole movement steps.

A particularly critical question to ponder is: “Do the statistical states uncovered by the HMM represent realized behavioural states that are relevant to the scale and scope of the research question?”. Based on our findings, we identify two key factors to consider in answering this question: (1) the extent to which behaviours exhibit distinct and separable movement signatures, and (2) the temporal scale of data collection.

First, compared to previous validation efforts in other taxa—such as seabirds [20, 23], African lions [22], and bats [21]—our results revealed a similar or even greater degree of mismatch between HMM-inferred and refence behaviours. Unlike species such as seabirds or some terrestrial carnivores that can exhibit clear distinctions in movement patterns across behavioural modes [10, 38–42], during summer, migratory caribou display considerable overlap in the video-detected behaviours across the nominal “stationary”, “foraging”, and “traveling” HMM states (Fig. 3; [13]). This behavioural complexity likely reduces the immediate interpretability of HMM states. For example, we found that in three-state models, the “foraging” state included all other behaviours – possibly because caribou, like many large herbivores, frequently multitask by feeding while stationary or in motion (Table S1; Figs. S3-S6; [43]). At fine temporal scales HMM states aligned well with certain caribou behaviours and can be applied to address specific objectives such as delineating stationary behaviours at a 20-minute scale, as the “stationary” state rarely included videos of caribou travelling or foraging. (Fig. 3). Although there was considerable overlap in video behaviours occurring in the “foraging” and “travelling” states of a three state HMM at 20-minute temporal scale, the improvement over a two-state model or coarser time resolutions was notable. We stress, however, that this apparent utility is contingent on careful framing: HMMs at this temporal scale may provide insights into broad contrasts in movement modes but should not be over-interpreted as validated biological states without further evidence.

Second, the temporal resolution of the data plays a critical role. We fitted HMMs to GPS data collected at 20-, 60-, and 120-minute intervals, whereas our reference videos captured behaviours in short 9–10 s clips. These clips provide only a brief snapshot of behaviour at the time of the GPS fix and may not fully reflect the dominant behaviour expressed across the entire relocation interval; in that sense this study is only a partial validation of behavioural inference. A resting clip could represent a brief pause in an otherwise extended foraging bout, or a feeding clip might capture the onset of a longer resting period. This temporal mismatch between short validation windows and longer GPS relocations contributed to the discrepancies we observed between video- and HMM-derived states, but this highlights the risk of assigning HMM states to the start points of observed steps. For example, a caribou could switch several times between resting, foraging, and traveling in the 120 min between GPS fixes (Table 1), and this likely explains why 20-minute HMM states align better with video behaviours than 120-minute HMM states. The disconnect between HMM-inferred states at classic monitoring temporal scales for ungulates and fine-scale animal behaviour presents a crucial consideration for conservation and management decisions that aim to use GPS data to identify critical habitat used for specific behaviours (e.g., foraging or birthing; [8, 11]).

Further underlining the problem of scale, we also found that HMM-inferred behavioural states, when assigned to the start points of observed steps, were inconsistent across temporal scales. This can create semantic confusion: a state labeled “traveling” at one scale may not correspond biologically to a “traveling” state at another despite sharing identical appellations. As Fryxell et al. [36] noted, large mobile animals can exhibit nested movement modes at different spatiotemporal scales. We therefore recommend that HMM-derived behavioural states be interpreted in relation to their temporal context, much like spatial scales are used to define orders of habitat selection [39]. For instance, HMM-derived behavioural inferences from GPS relocations collected at daily intervals could potentially capture broad-scale migratory versus non-migratory states, whereas ≤ 5-minute intervals may be necessary to detect localized behaviours such as foraging or resting.

## Conclusions

Our results are a reminder that although HMMs are a powerful statistical tool to cluster tracks into phases with similar movement patterns, their interpretation in terms of behavioural states depends highly on observed variables and sampling intervals. Across all models, HMMs effectively separated fast, directed movement from slow, tortuous movement based on step lengths and turning angles at given temporal scales, but these states did not consistently align with actual behaviours observed on short videos at given GPS positions. The reliability of HMM behavioural inferences is likely highly contingent on the intricacy of movement patterns which varies across species, populations, individuals, ages, and sexes [44–47]. Furthermore, these patterns may shift over time—such as across diurnal or seasonal cycles [48, 49].

We stress that robust behavioural inference from HMMs requires deep ecological knowledge of the study system as emphasized by McClintock et al. [25]. Decisions such as the selection of fix rates or the resampling of data to coarser temporal scales to detect specific behavioural states will determine the inference that can be gleaned from HMMs. A key consideration when deciding on fix rates and HMM implementation is the true duration of the specific behaviours of interest; for example, an observational study of fine-scale caribou behaviours (e.g., lying, standing, trotting) reported much shorter mean behavioural bouts (0–53 min; [50]) than a study which also used observational methods but delineated behaviours only into resting or active categories (70–275 min; [51]).

Greater interpretability of HMM behavioural states might be achieved by using finer temporal scales and/or integrating complementary data sources such as accelerometry and activity sensors [52–54]. Stronger validation than what was done in our study could also be achieved by increasing the number of video clips per interval to approximate the average behaviour expressed over the relocation interval, or ideally, by collecting continuous behavioural observations that capture the predominant behaviours throughout the entire interval. To advance the field of movement ecology, we encourage further validation of HMM-inferred behavioural states across a wider range of species, temporal scales, and data types. By presenting this simplified validation, our aim is to spark critical reflection on the risks of naïve HMM applications and to highlight the importance of cautious, well-informed interpretation and validation.

## Supplementary Information

Below is the link to the electronic supplementary material.

Supplementary Material 1

## Data Availability

All R code used to generate results and figures is available at the following OSF link (https://osf.io/zxtvw/?view_only=75b567324b5245168cdebff87e9b6643). The datasets generated and analyzed for this research are sensitive and not publicly available. Animal location data are owned by the State of Alaska, Government of Yukon, and Caribou Ungava. Data can be made available upon request for qualified researchers.

## Abbreviations

HMM: Stands for Hidden Markov Model
FMCH: Stands for Fortymile Caribou Herd
RFCH: Stands for Rivière-aux-Feuilles Caribou Herd

## References

[1] Nathan R, Getz WM, Revilla E, Holyoak M, Kadmon R, Saltz D, et al. A movement ecology paradigm for unifying organismal movement research. Proc Natl Acad Sci. 2008;105(49):19052–59. 10.1073/pnas.0800375105 PMC2614714 19060196

[2] Kays R, Crofoot MC, Jetz W, Wikelski M. Terrestrial animal tracking as an eye on life and planet. Science. 2015;348(6240):aaa2478. 26068858 10.1126/science.aaa2478

[3] Wilmers CC, Nickel B, Bryce CM, Smith JA, Wheat RE, Yovovich V. The golden age of bio-logging: how animal‐borne sensors are advancing the frontiers of ecology. Ecology. 2015;96(7):1741–53. 26378296 10.1890/14-1401.1

[4] Patterson TA, Thomas L, Wilcox C, Ovaskainen O, Matthiopoulos J. State–space models of individual animal movement. Trends Ecol Evol. 2008;23(2):87–94. 18191283 10.1016/j.tree.2007.10.009

[5] Gurarie E, Fleming CH, Fagan WF, Laidre KL, Hernández-Pliego J, Ovaskainen O. Correlated velocity models as a fundamental unit of animal movement: synthesis and applications. Mov Ecol. 2017;5(13):1–18. 28496983 10.1186/s40462-017-0103-3 PMC5424322

[6] Langrock R, King R, Matthiopoulos J, Thomas L, Fortin D, Morales JM. Flexible and practical modeling of animal telemetry data: hidden Markov models and extensions. Ecology. 2012;93(11):2336–42. 23236905 10.1890/11-2241.1

[7] Beumer LT, Pohle J, Schmidt NM, Chimienti M, Desforges JP, Hansen LH, et al. An application of upscaled optimal foraging theory using hidden Markov modelling: year-round behavioural variation in a large Arctic herbivore. Mov Ecol. 2020;8(5):1–16. 32518653 10.1186/s40462-020-00213-x PMC7275509

[8] Brushett A, Whittington J, Macbeth B, Fryxell JM. Changes in movement, habitat use, and response to human disturbance accompany parturition events in Bighorn sheep (*Ovis canadensis*). Mov Ecol. 2023;11(1):36. 37403172 10.1186/s40462-023-00404-2 PMC10318713

[9] Couriot OH, Cameron MD, Joly K, Adamczewski J, Campbell MW, Davison T, et al. Continental synchrony and local responses: Climatic effects on Spatiotemporal patterns of calving in a social ungulate. Ecosphere. 2023;14(1):e4399.

[10] Whittington J, Hebblewhite M, Baron RW, Ford AT, Paczkowski J. Towns and trails drive carnivore movement behaviour, resource selection, and connectivity. Mov Ecol. 2022;10(1):17. 35395833 10.1186/s40462-022-00318-5 PMC8994267

[11] Festa-Bianchet M, Ray JC, Boutin S, Côté SD, Gunn A. Conservation of caribou (*Rangifer tarandus*) in Canada: an uncertain future. Can J Zool. 2011;89(5):419–34.

[12] Stokes KL, Broderick AC, Canbolat AF, Candan O, Fuller WJ, Glen F, et al. Migratory corridors and foraging hotspots: critical habitats identified for Mediterranean green turtles. Divers Distrib. 2015;21(6):665–74.

[13] Ehlers L, Palm E, Herriges J, Bentzen T, Suitor M, Joly K, et al. A taste of space: remote animal observations and discrete-choice models provide new insights into foraging and density dynamics for a large Subarctic herbivore. J Anim Ecol. 2024;93(7):891–05. 38773852 10.1111/1365-2656.14109

[14] Zucchini W, MacDonald IL. Hidden Markov models for time series: an introduction using R. Chapman and Hall/CRC. 2009.

[15] Michelot T, Langrock R, Patterson TA. MoveHMM: an R package for the statistical modelling of animal movement data using hidden Markov models. Methods Ecol Evol. 2016;7(11):1308–15.

[16] Morales JM, Haydon DT, Frair J, Holsinger KE, Fryxell JM. Extracting more out of relocation data: Building movement models as mixtures of random walks. Ecology. 2004;85(9):2436–45.

[17] Pohle J, Langrock R, Van Beest FM, Schmidt NM. Selecting the number of States in hidden Markov models: pragmatic solutions illustrated using animal movement. J Agric Biol Environ Stat. 2017;22:270–93.

[18] Prima MC, Duchesne T, Merkle JA, Chamaillé-Jammes S, Fortin D. Multi-mode movement decisions across widely ranging behavioral processes. PLoS ONE. 2022;17(8):e0272538. 35951664 10.1371/journal.pone.0272538 PMC9371300

[19] Bacheler NM, Michelot T, Cheshire RT, Shertzer KW. Fine-scale movement patterns and behavioral States of Gray triggerfish *Balistes Capriscus* determined from acoustic telemetry and hidden Markov models. Fish Res. 2019;215:76–89.

[20] Bennison A, Bearhop S, Bodey TW, Votier SC, Grecian WJ, Wakefield ED, et al. Search and foraging behaviors from movement data: a comparison of methods. Ecol Evol. 2018;8(1):13–24. 29321847 10.1002/ece3.3593 PMC5756868

[21] Hurme E, Gurarie E, Greif S, Herrera MLG, Flores-Martínez JJ, Wilkinson GS, et al. Acoustic evaluation of behavioral States predicted from GPS tracking: a case study of a marine fishing Bat. Mov Ecol. 2019;7(21):1–14. 31223482 10.1186/s40462-019-0163-7 PMC6567457

[22] Goodall VL, Ferreira SM, Funston PJ, Maruping-Mzileni N. Uncovering hidden States in African Lion movement data using hidden Markov models. Wildl Res. 2019;46(4):296–03.

[23] Akeresola RA, Butler A, Jones EL, King R, Elvira V, Black J, et al. Validating hidden Markov models for seabird behavioural inference. Ecol Evol. 2024;14(3):e11116. 38440082 10.1002/ece3.11116 PMC10911961

[24] Augusiak J, Van den Brink PJ, Grimm V. Merging validation and evaluation of ecological models to ‘evaludation’: a review of terminology and a practical approach. Ecol Modell. 2014;280:117–28.

[25] McClintock BT, Langrock R, Gimenez O, Cam E, Borchers DL, Glennie R, Patterson T. A. Uncovering ecological state dynamics with hidden Markov models. Ecol Lett. 2020;23(12):1878–903. 33073921 10.1111/ele.13610 PMC7702077

[26] Glennie R, Adam T, Leos-Barajas V, Michelot T, Photopoulou T, McClintock BT. Hidden Markov models: pitfalls and opportunities in ecology. Methods Ecol Evol. 2023;14(1):43–56.

[27] Johnson DH. The comparison of usage and availability measurements for evaluating resource preference. Ecology. 1980;61(1):65–71.

[28] Heaslip SG, Iverson SJ, Bowen WD, James MC. Jellyfish support high energy intake of leatherback sea turtles (*Dermochelys coriacea*): video evidence from animal-borne cameras. PLoS ONE. 2012;7(3):e33259. 22438906 10.1371/journal.pone.0033259 PMC3306388

[29] Thompson ID, Bakhtiari M, Rodgers AR, Baker JA, Fryxell JM, Iwachewski E. Application of a high-resolution animal‐borne remote video camera with global positioning for wildlife study: observations on the secret lives of woodland caribou. Wildl Soc Bull. 2012;36(2):365–70.

[30] Patel A, Stocks B, Fisher C, Nicolls F, Boje E. Tracking the cheetah tail using animal-borne cameras, GPS, and an IMU. IEEE Sens Lett. 2017;1(4):1–4.

[31] Ehlers L, Coulombe G, Herriges J, Bentzen T, Suitor M, Joly K, et al. Critical summer foraging tradeoffs in a Subarctic ungulate. Ecol Evol. 2021;11(24):17835–72. 35003643 10.1002/ece3.8349 PMC8717276

[32] Béland S, Vuillaume B, Leclerc M, Bernier M, Côté SD. Selection of summer feeding sites and food resources by female migratory caribou (*Rangifer tarandus*) determined using camera collars. PLoS ONE. 2023;18(11):e0294846. 38019854 10.1371/journal.pone.0294846 PMC10686509

[33] R Core Team. R: A Language and Environment for Statistical Computing_. R Foundation for Statistical Computing, Vienna, Austria. 2024. https://www.R-project.org/

[34] Signer J, Fieberg J, Avgar T. Animal movement tools (amt): R package for managing tracking data and conducting habitat selection analyses. Ecol Evol. 2019;9(2):880–90. 30766677 10.1002/ece3.4823 PMC6362447

[35] McClintock BT. Worth the effort? A practical examination of random effects in hidden Markov models for animal telemetry data. Methods Ecol Evol. 2021;12(8):1475–97.

[36] Fryxell JM, Hazell M, Börger L, Dalziel BD, Haydon DT, Morales JM, et al. Multiple movement modes by large herbivores at multiple Spatiotemporal scales. Proc Natl Acad Sci. 2008;105(49):19114–19. 10.1073/pnas.0801737105 PMC2614724 19060190

[37] Kuhn M. Building predictive models in R using the caret package. J Stat Softw. 2008;28(5):1–26.27774042

[38] Dragon AC, Bar-Hen A, Monestiez P, Guinet C. Comparative analysis of methods for inferring successful foraging areas from Argos and GPS tracking data. Mar Ecol Prog Ser. 2012;452:253–67.

[39] Dean B, Freeman R, Kirk H, Leonard K, Phillips RA, Perrins CM, et al. Behavioural mapping of a pelagic seabird: combining multiple sensors and a hidden Markov model reveals the distribution of at-sea behaviour. J R Soc Interface. 2013;10(78):20120570. 23034356 10.1098/rsif.2012.0570 PMC3565783

[40] Miller MG, Carlile N, Phillips JS, McDuie F, Congdon BC. Importance of tropical tuna for seabird foraging over a marine productivity gradient. Mar Ecol Prog Ser. 2018;586:233–49.

[41] van Beest FM, Mews S, Elkenkamp S, Schuhmann P, Tsolak D, Wobbe T, et al. Classifying grey seal behaviour in relation to environmental variability and commercial fishing activity-a multivariate hidden Markov model. Sci Rep. 2019;9(1):5642. 30948786 10.1038/s41598-019-42109-w PMC6449369

[42] Zhang J, Rayner M, Vickers S, Landers T, Sagar R, Stewart J, et al. GPS telemetry for small seabirds: using hidden Markov models to infer foraging behaviour of common diving petrels (*Pelecanoides urinatrix urinatrix*). Emu. 2019;119(2):126–37.

[43] Fortin D, Boyce MS, Merrill EH. Multi-tasking by mammalian herbivores: overlapping processes during foraging. Ecology. 2004;85(8):2312–22.

[44] Bates LA, Byrne RW. Sex differences in the movement patterns of free-ranging chimpanzees (*Pan troglodytes schweinfurthii*): foraging and border checking. Behav Ecol Sociobiol. 2009;64:247–55.

[45] Mueller T, Olson KA, Dressler G, Leimgruber P, Fuller TK, Nicolson C, et al. How landscape dynamics link individual-to population‐level movement patterns: a multispecies comparison of ungulate relocation data. Glob Ecol Biogeogr. 2011;20(5):683–94.

[46] Speed CW, Meekan MG, Field IC, McMahon CR, Stevens JD, McGregor F, et al. Spatial and Temporal movement patterns of a multi-species coastal reef shark aggregation. Mar Ecol Prog Ser. 2011;429:261–75.

[47] Webber QM, Laforge MP, Bonar M, Robitaille AL, Hart C, Zabihi-Seissan S, et al. The ecology of individual differences empirically applied to space-use and movement tactics. Am Nat. 2020;196(1):1–15. 32552106 10.1086/708721

[48] Van Beest FM, Vander Wal E, Stronen AV, Brook RK. Factors driving variation in movement rate and seasonality of sympatric ungulates. J Mammal. 2013;94(3):691–01.

[49] Ensing EP, Ciuti S, de Wijs FA, Lentferink DH, Ten Hoedt A, Boyce MS, et al. GPS based daily activity patterns in European red deer and North American elk (*Cervus elaphus*): indication for a weak circadian clock in ungulates. PLoS ONE. 2014;9(9):e106997. 25208246 10.1371/journal.pone.0106997 PMC4160215

[50] Boertje RD. Seasonal activity of the Denali caribou herd, Alaska. Rngifer. 1985;5(2):32–42.

[51] Maier JA, White RG. Timing and synchrony of activity in caribou. Can J Zool. 1998;76(11):1999–2009.

[52] Guo Y, Poulton G, Corke P, Bishop-Hurley GJ, Wark T, Swain DL. Using accelerometer, high sample rate GPS and magnetometer data to develop a cattle movement and behaviour model. Ecol Model. 2009;220(17):2068–75.

[53] Adam T, Griffiths CA, Leos-Barajas V, Meese EN, Lowe CG, Blackwell PG, Langrock R. Joint modelling of multi‐scale animal movement data using hierarchical hidden Markov models. Methods Ecol Evol. 2019;10(9):1536–50.

[54] Chimienti M, van Beest FM, Beumer LT, Desforges JP, Hansen LH, Stelvig M, et al. Quantifying behavior and life-history events of an Arctic ungulate from year‐long continuous accelerometer data. Ecosphere. 2021;12(6):e03565.

