# Supplementary material for: Inferring behavioural states from tracking data with hidden Markov models – a validation study using GPS video-camera collars
Source: Mov Ecol. 2026 Jan 24;14:11. doi: 10.1186/s40462-025-00621-x (PMC12924397; doi:10.1186/s40462-025-00621-x)
Supplement: Supplementary file 1 — Supplementary Material 1 [file 40462_2025_621_MOESM1_ESM.docx]

Supplementary material for

Inferring behavioral states from tracking data with hidden Markov models – A validation study using GPS video-camera collars

Benjamin Larue, Jonathan J. Farr, Libby Ehlers, Jim Herriges, Torsten Bentzen, Michael J. Suitor, Kyle Joly, Théo Michelot, Barbara Villaume, Steeve D. Côté, Eliezer Gurarie, and Mark Hebblewhite

Corresponding author: [benjamin.larue@umt.edu](mailto:benjamin.larue@umt.edu)

**This PDF file includes:**

Supplementary material S1: Supplementary table and figures

Supplementary material S1:

Supplementary table and figures

**Table S1.** Percentage of video collar behaviours in each of the two and three-state HMMs based on 20-, 60-, and 120-minute relocation intervals from the Fortymile Caribou herd.

| HMM | GPS  interval | HMM-inferred  behavioural state | Stationary Awake | Ruminating | Napping | Eating | Travelling |
| --- | --- | --- | --- | --- | --- | --- | --- |
| 2-state | 20 | Stationary | 16.0 | 36.2 | 8.3 | 35.4 | 4.1 |
|  |  | Travelling | 4.6 | 10.7 | 0.6 | 55.7 | 28.4 |
|  | 60 | Stationary | 13.5 | 29.5 | 6.5 | 43.2 | 7.3 |
|  |  | Travelling | 5.3 | 16.4 | 1.2 | 47.6 | 29.4 |
|  | 120 | Stationary | 13.2 | 29.3 | 6.5 | 43.3 | 7.7 |
|  |  | Travelling | 7.9 | 21.8 | 3.0 | 45.4 | 21.9 |
| 3-state | 20 | Stationary | 21.4 | 54.2 | 16.1 | 7.7 | 0.5 |
|  |  | Foraging | 10.7 | 20.8 | 2.4 | 57.5 | 8.6 |
|  |  | Travelling | 3.6 | 9.4 | 0.2 | 52.0 | 34.8 |
|  | 60 | Stationary | 15.0 | 32.8 | 7.6 | 40.2 | 4.3 |
|  |  | Foraging | 8.9 | 21.4 | 3.2 | 50.4 | 16.1 |
|  |  | Travelling | 3.0 | 11.2 | 0.1 | 42.5 | 43.2 |
|  | 120 | Stationary | 14.8 | 29.0 | 8.4 | 42.8 | 5.1 |
|  |  | Foraging | 11.5 | 28.5 | 5.2 | 44.5 | 10.4 |
|  |  | Travelling | 7.7 | 19.5 | 2.6 | 44.2 | 26.1 |

Table S2. Step length and turn angle parameters derived from hidden-Markov models of GPS relocation data for the Fortymile caribou herd (FMCH) and Rivière-aux-Feuilles caribou herd (RFCH).

| Population | GPS  Relocation Interval | Model states | Parameters | Stationary state | Foraging  state | Travelling state |
| --- | --- | --- | --- | --- | --- | --- |
| FMCH | 20 | 2 | Step mean | 0.037 | - | 0.411 |
|  |  |  | Step std. dev. | 0.049 | - | 0.324 |
|  |  |  | Angle mean | -3.059 | - | -0.004 |
|  |  |  | Angle concentration | 0.043 | - | 2.358 |
|  | 60 |  | Step mean | 0.227 | - | 1.21 |
|  |  |  | Step std. dev. | 0.298 | - | 0.907 |
|  |  |  | Angle mean | -0.032 | - | -0.007 |
|  |  |  | Angle concentration | 0.258 | - | 3.32 |
|  | 120 |  | Step mean | 0.423 | - | 1.723 |
|  |  |  | Step std. dev. | 0.485 | - | 1.478 |
|  |  |  | Angle mean | -0.054 | - | -0.013 |
|  |  |  | Angle concentration | 0.245 | - | 2.99 |
|  | 20 | 3 | Step mean | 0.003 | 0.089 | 0.519 |
|  |  |  | Step std. dev. | 0.002 | 0.091 | 0.333 |
|  |  |  | Angle mean | -3.134 | -0.012 | -0.002 |
|  |  |  | Angle concentration | 0.557 | 0.517 | 3.183 |
|  | 60 |  | Step mean | 0.111 | 0.614 | 1.791 |
|  |  |  | Step std. dev. | 0.141 | 0.512 | 0.859 |
|  |  |  | Angle mean | -0.171 | -0.007 | -0.014 |
|  |  |  | Angle concentration | 0.04 | 1.311 | 5.35 |
|  | 120 |  | Step mean | 0.163 | 0.719 | 2.023 |
|  |  |  | Step std. dev. | 0.176 | 0.733 | 1.603 |
|  |  |  | Angle mean | -2.718 | 0.005 | -0.021 |
|  |  |  | Angle concentration | 0.089 | 0.708 | 4.402 |
| RFCH | 60 | 2 | Step mean | 0.181 | - | 1.326 |
|  |  |  | Step std. dev. | 0.262 | - | 1.015 |
|  |  |  | Angle mean | 0.024 | - | -0.005 |
|  |  |  | Angle concentration | 0.144 | - | 4.156 |
|  | 120 |  | Step mean | 0.373 | - | 2.017 |
|  |  |  | Step std. dev. | 0.486 | - | 1.795 |
|  |  |  | Angle mean | -0.023 | - | -0.005 |
|  |  |  | Angle concentration | 0.179 | - | 4.478 |
|  | 60 | 3 | Step mean | 0.065 | 0.55 | 1.766 |
|  |  |  | Step std. dev. | 0.088 | 0.526 | 0.99 |
|  |  |  | Angle mean | 3.127 | 0.002 | -0.007 |
|  |  |  | Angle concentration | 0.125 | 1.16 | 7.773 |
|  | 120 |  | Step mean | 0.159 | 0.692 | 2.277 |
|  |  |  | Step std. dev. | 0.187 | 0.846 | 1.837 |
|  |  |  | Angle mean | -3.055 | -0.007 | -0.005 |
|  |  |  | Angle concentration | 0.059 | 0.645 | 6.973 |


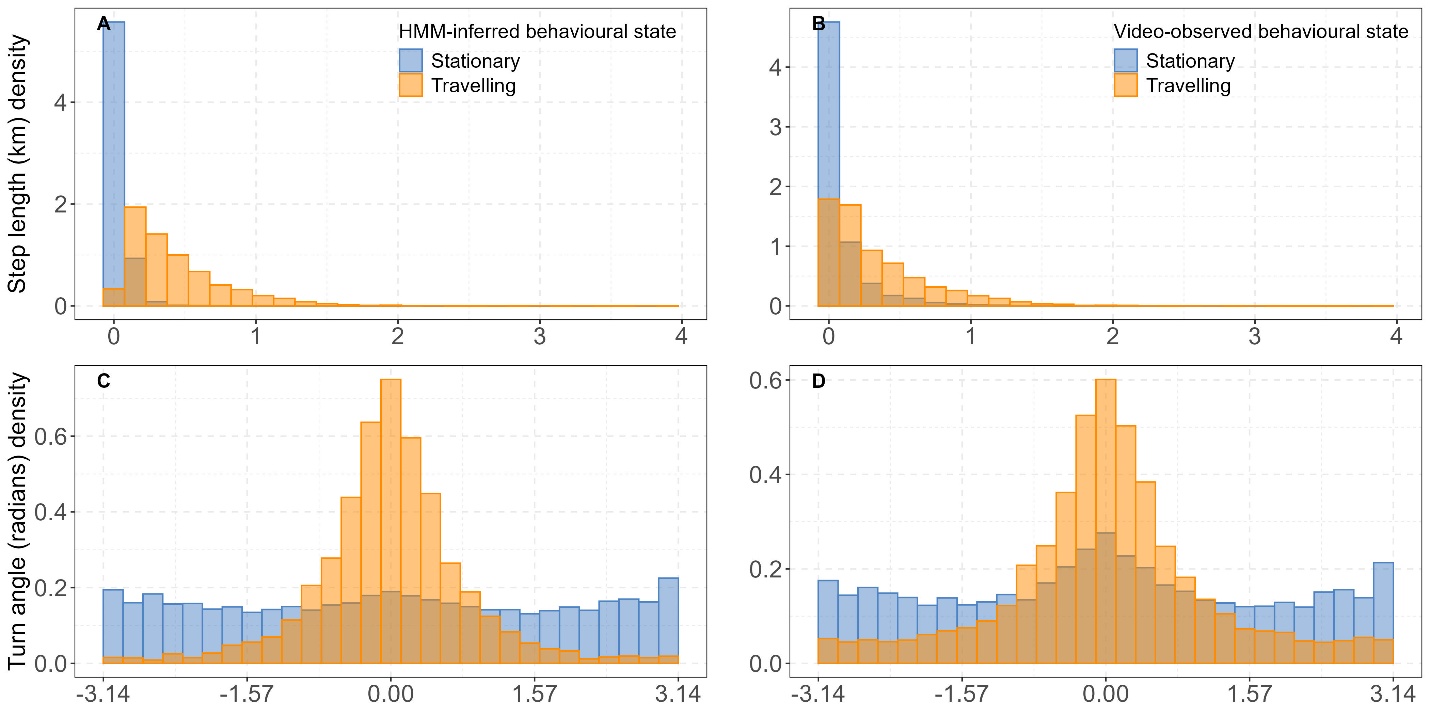


**Fig. S1.** Hidden Markov model (HMM) inferred and video-observed behavioural classifications from Fortymile caribou herd GPS video-camera collar data resampled to 20-minute fix intervals. HMM-inferred behavioural states are classified from a two-state HMM using the Viterbi algorithm, and video behaviours are classified by trained observers. The top row shows among-behaviour heterogeneity in step length distributions for HMM classifications (A) and video behaviour classifications (B). The bottom row shows among-behaviour heterogeneity in turn angle distributions for HMM classifications (C) and video behaviour classifications (D).


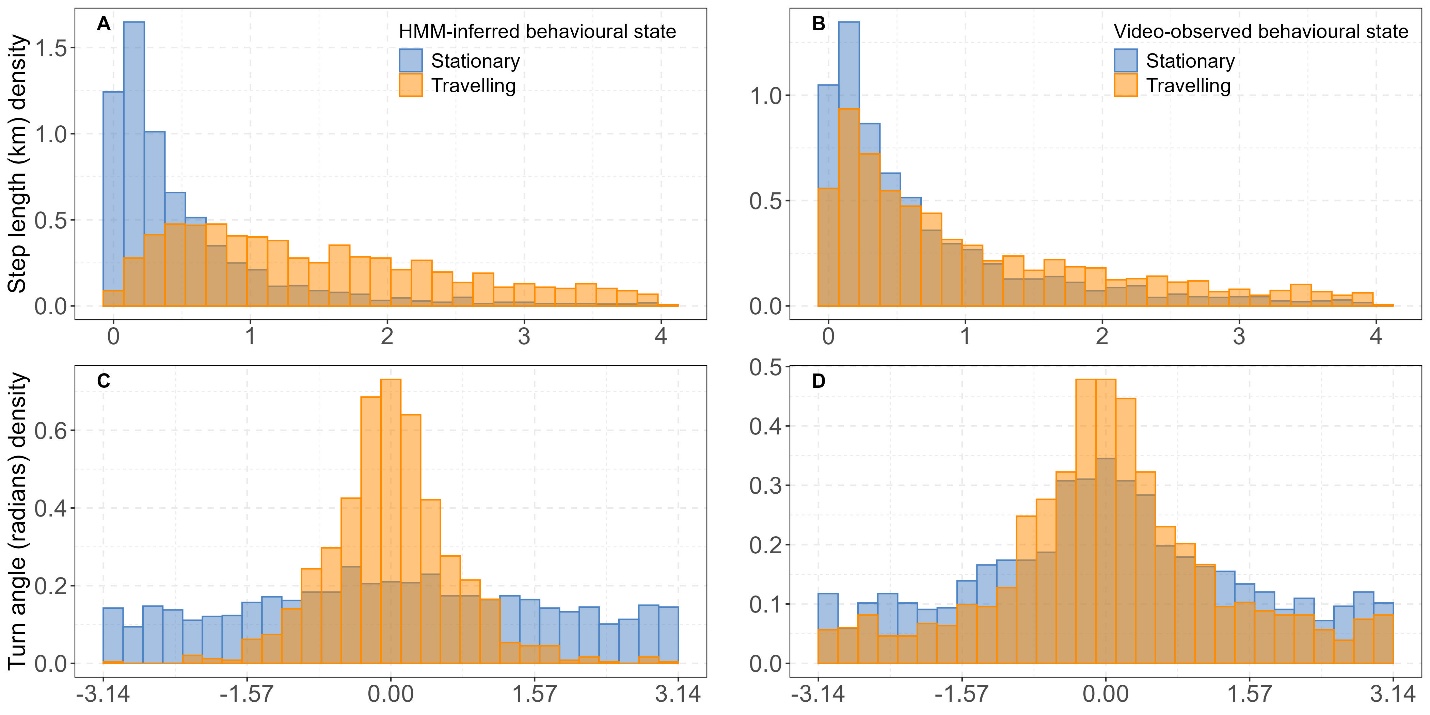


**Fig. S2.** Hidden Markov model (HMM) inferred and video-observed behavioural classifications from Fortymile caribou herd GPS video-camera collar data resampled to 120-minute fix intervals. HMM-inferred behavioural states are classified from a two-state HMM using the Viterbi algorithm, and video behaviours are classified by trained observers. The top row shows among-behaviour heterogeneity in step length distributions for HMM classifications (A) and video behaviour classifications (B). The bottom row shows among-behaviour heterogeneity in turn angle distributions for HMM classifications (C) and video behaviour classifications (D).


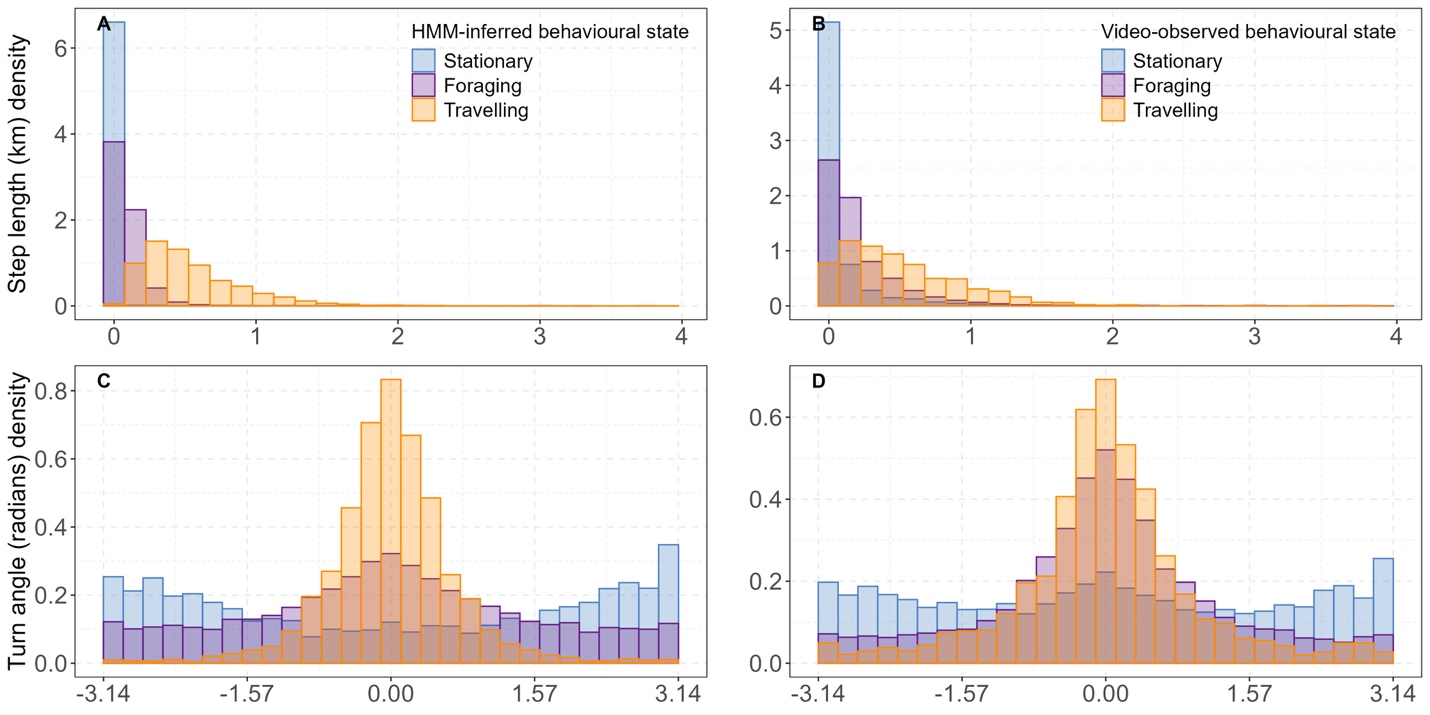


**Fig. S3.** Hidden Markov model (HMM) inferred and video-observed behavioural classifications from Fortymile caribou herd GPS video-camera collar data resampled to 20-minute fix intervals. HMM-inferred behavioural states are classified from a three-state HMM using the Viterbi algorithm, and video behaviours are classified by trained observers. The top row shows among-behaviour heterogeneity in step length distributions for HMM classifications (A) and video behaviour classifications (B). The bottom row shows among-behaviour heterogeneity in turn angle distributions for HMM classifications (C) and video behaviour classifications (D).


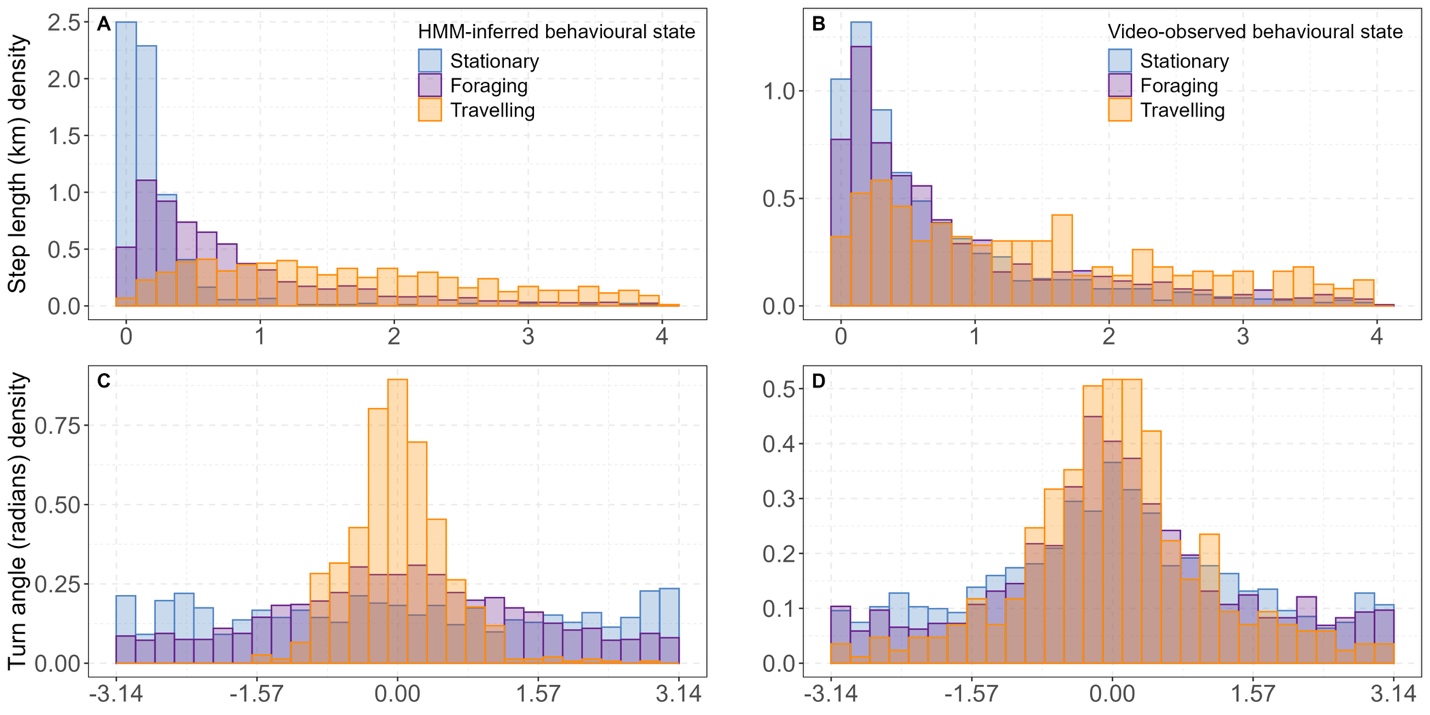


**Fig. S4.** Hidden Markov model (HMM) inferred and video-observed behavioural classifications from Fortymile caribou herd GPS video-camera collar data resampled to 120-minute fix intervals. HMM-inferred behavioural states are classified from a three-state HMM using the Viterbi algorithm, and video behaviours are classified by trained observers. The top row shows among-behaviour heterogeneity in step length distributions for video behaviour classifications (A) and HMM classifications (B). The bottom row shows among-behaviour heterogeneity in turn angle distributions for video behaviour classifications (C) and HMM classifications (D).


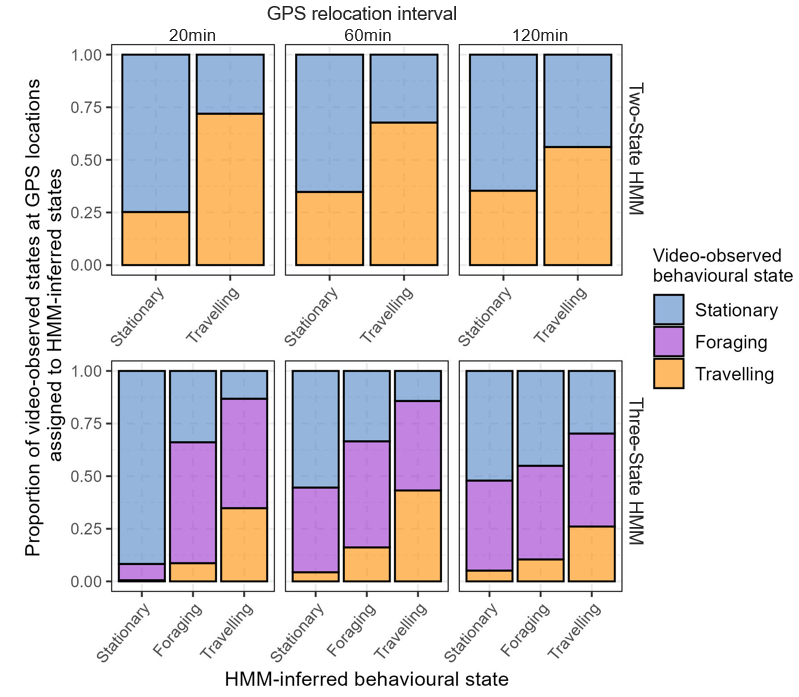


**Fig. S5.** Contingency mosaic presenting the proportion of Fortymile caribou video-observed behavioural classifications in each inferred hidden Markov model (HMM) Viterbi behavioural state in two-state models (top row) and three-state models (bottom row) at different GPS relocation intervals (from left to right: 20 min, 60 min, and 120 min).


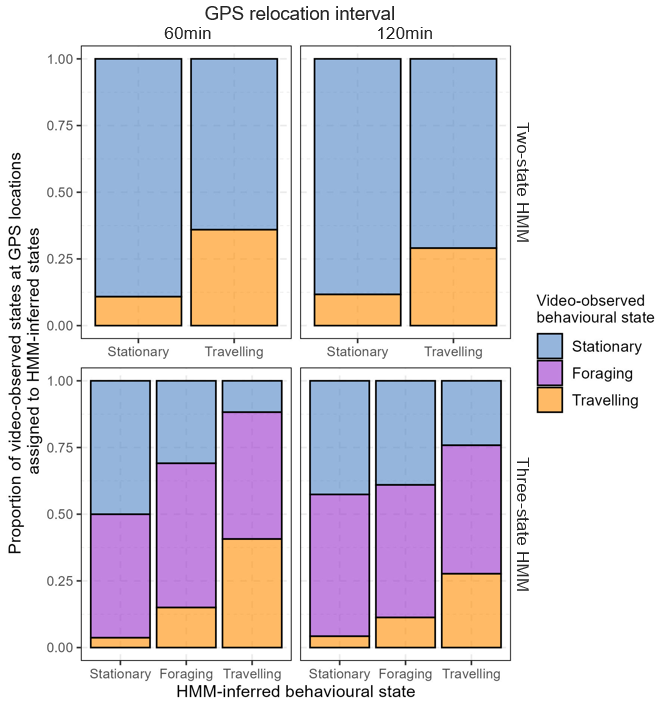


**Fig. S6.** Contingency mosaic presenting the proportion of Rivière-aux-feuilles caribou video-observed behavioural classifications in each inferred hidden Markov model (HMM) Viterbi behavioural state in two-state models (top row) and three-state models (bottom row) at different GPS relocation intervals (from left to right: 60 min, and 120 min).


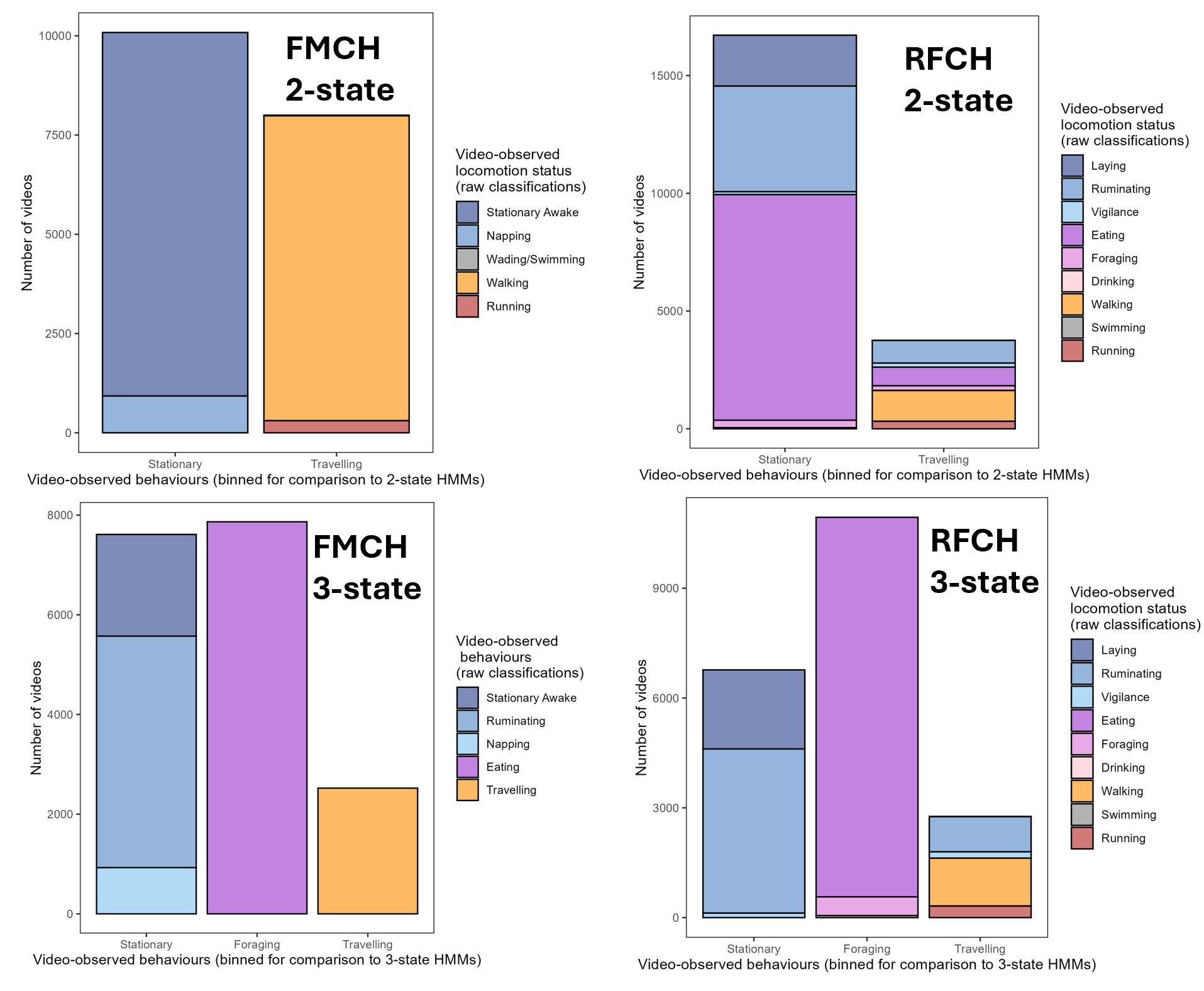


**Fig S7.** Raw video-observed behaviours and locomotion status for two caribou populations binned into two or three categories for comparison to hidden-Markov model (HMM) derived movement states. Full details on video re-classification into HMM states are provided in the methods section.
